# Translation-Associated Mutational U-Pressure in the First ORF of SARS-CoV-2 and Other Coronaviruses

**DOI:** 10.3389/fmicb.2020.559165

**Published:** 2020-09-22

**Authors:** Vladislav Victorovich Khrustalev, Rajanish Giri, Tatyana Aleksandrovna Khrustaleva, Shivani Krishna Kapuganti, Aleksander Nicolaevich Stojarov, Victor Vitoldovich Poboinev

**Affiliations:** ^1^Department of General Chemistry, Belarusian State Medical University, Minsk, Belarus; ^2^School of Basic Sciences, Indian Institute of Technology Mandi, Mandi, India; ^3^Biochemical Group of Multidisciplinary Diagnostic Laboratory, Institute of Physiology of the National Academy of Sciences of Belarus, Minsk, Belarus; ^4^Department of Radiation Medicine and Ecology, Belarusian State Medical University, Minsk, Belarus

**Keywords:** COVID-19, SARS, MERS, mutational pressure, cytosine deamination

## Abstract

Within 4 months of the ongoing COVID-19 pandemic caused by SARS-CoV-2, more than 250 nucleotide mutations have been detected in ORF1ab of the virus isolated from infected persons from different parts of the globe. These observations open up an obvious question about the rate and direction of mutational pressure for further vaccine and therapeutics designing. In this study, we did a comparative analysis of ORF1a and ORF1b by using the first isolate (Wuhan strain) as the parent sequence. We observed that most of the nucleotide mutations are C to U transitions. The rate of synonymous C to U transitions is significantly higher than the rate of non-synonymous ones, indicating negative selection on amino acid substitutions. Further, trends in nucleotide usage bias have been investigated in 49 coronaviruses species. A strong bias in nucleotide usage in fourfold degenerate sites toward uracil residues is seen in ORF1ab of all the studied coronaviruses: both in the ORF1a and in the ORF1b translated thanks to the programmed ribosomal frameshifting that has an efficiency of 14 – 45% in different species. A more substantial mutational U-pressure is observed in ORF1a than in ORF1b perhaps because ORF1a is translated more frequently than ORF1b. Mutational U-pressure is there even in ORFs that are not translated from genomic RNA plus strands, but the bias is weaker than in ORF1ab. Unlike other nucleotide mutations, mutational U-pressure caused by cytosine deamination, mostly occurring during the RNA plus strand replication and also translation, cannot be corrected by the proof-reading machinery of coronaviruses. The knowledge generated on the mutational U-pressure that becomes stronger during translation of viral RNA plus strands has implications for vaccine and nucleoside analog development for treating COVID-19 and other coronavirus infections.

## Introduction

The current COVID-19 pandemic caused by SARS-CoV-2 has claimed more than 0.25 million human lives with nearly 3.5 million reported infections globally and still this number is increasing day by day (WHO, 2020). The virus was first detected in a wet market in Wuhan, China, in December 2019. It bears a close similarity to bat coronaviruses, and similar viral species have been detected in pangolin. This led to the speculation that the virus may have originated in bats with pangolins acting as intermediate hosts. Since then, the virus has spread and infected people in each and every country. The infections are characterized by sore throat, fever, cough, body pains, breathlessness, severe pneumonia, and death due to multi-organ failure involving kidneys and lungs (Perlman and Netland, 2009; Lauber et al., 2012).

Coronaviruses have an exceptionally large genome of around 30 kb. A huge part of the genome, at the 5′ end, around 20 kb, harbors a replicase gene that codes for around 16 non-structural proteins (nsps) that are cleaved from the long precursor (Snijder et al., 2016). The rest of the genome, near the 3′ end, codes for the structural (spike, envelope, membrane, and nucleocapsid) and several accessory proteins (Nga et al., 2011). The replicase gene (ORF1ab), according to numerous descriptions in GenBank, includes two subunits, ORF1a and ORF1ab expressing the polyproteins pp1a and pp1ab, respectively. A slippery sequence and ribosomal frameshifting caused by an RNA pseudoknot ensure the translation of both polyproteins from the same genome (Brierley et al., 1989; Baranov et al., 2005). Actually, ORF1b is never translated alone, but only as a part of ORF1ab if frameshifting is successful. If frameshifting is not successful, once ORF1a is translated, ribosome reaches the terminal codon and dissociates from RNA. Pp1a has nsps 1–11 whereas pp1ab has nsps 1–16 (Ziebuhr et al., 2000). The nsps form the viral replicase complexes. Here both genomic and subgenomic viral RNAs get synthesized through negative-strand intermediates. Subgenomic RNAs code for structural and accessory proteins. Those proteins are not translated from the genomic RNA plus strand, but only from subgenomic RNAs.

Variations in viral sequences have an important role in viral propagation and pathogenesis. They may help change the viral phenotype allowing it to evade host immune system and also acquire drug resistance. These variations may arise from copying errors during genome replication (Bass, 2002). RNA viruses have low replicative fidelity; hence are more prone to mutations than DNA viruses. Around 10^–4^ to 10^–6^ errors per nucleotide are seen in RNA viruses, which is equivalent to approximately one mutation per genome per replication cycle. This allows the viruses to maintain mutations that are beneficial for them in adapting to new environments (Sanjuan et al., 2010). Sometimes, a specific nucleotide mutation occurs more frequently than others, which is called directional mutational pressure. Mutational pressure changes the nucleotide composition of the genome irreversibly and may be caused by different factors: by error-prone polymerase, by RNA editing, by oxidative damage to amine bases. Moreover, the direction of mutational pressure may not be similar along the entire length of the genome (Khrustalev et al., 2017). Coronaviruses are known as slowly mutating RNA viruses since they perform proof-reading during replication (Bouvet et al., 2012).

Cytosine to Uracil transitions can occur spontaneously through oxidative damage by free radicals or enzymatically through the action of apolipoprotein B mRNA editing catalytic subunit (APOBEC) family of cytidine deaminases. RNA-editing enzymes from APOBEC family bind single-stranded viral RNA and deaminate cytosine residues (Sharma and Baysal, 2017; Smith, 2017). Single nucleotide transitions mostly result in synonymous mutations – though synonymous codons, i.e., codons that code for same amino acid, don’t occur equally in different organisms; these organisms are said to have a codon usage bias. Fourfold degenerate codons can tolerate any nucleotide substitution at the wobble or the third position, which doesn’t change the amino acid sequence, but just contributes into the codon usage bias. But even a single changed nucleotide in the RNA may sometimes affect the RNA structure and interference (Khrustalev et al., 2017).

The life cycle of coronaviruses is rather complicated. After entering the cell, the process of genomic RNA plus strand translation begins and usually (at least, for SARS-CoV-1) ends at the stop codon of ORF1a (the ribosome/RNA complex is disassembled). Sometimes the ribosome slips into a new reading frame at a programmed ribosomal frameshift sequence and translation of ORF1ab proceeds until the stop codon of the ORF1b. It means that ORF1b is translated less frequently than ORF1a, while the rest of the RNA is not translated at all. Before translation, the RNA plus strand exists as a complex of folded RNA with viral nucleoprotein. Single stranded RNA forms secondary structure due to canonical and non-canonical intramolecular base pairing (Sato et al., 2009). These stem-loops and G-quadruplexes must be unwound before the translation. Both translation factors [with RNA helicase activity (Fidaleo et al., 2016; Díaz-López et al., 2019)] and ribosome itself are able to unwind mRNA (Xie and Chen, 2017). It means that during translation RNA exists in a single-stranded (unwound) state and in that moment its amine bases can be damaged by oxidative agents at much higher probability than in the non-translated state. According to our hypothesis, the higher the rate of translation for a region of genomic RNA-plus strand, the stronger should be the mutational pressure (and nucleotide usage bias) inside it.

Since its first appearance in December, 2019, four hundred full-length sequences of ORF1ab of SARS-CoV-2 have become available on GenBank. In this study, a comparative analysis of ORF1a and ORF1b has been carried out, taking the first isolate (GenBank ID: NC_045512) as the parent sequence. Further, trends in nucleotide usage bias have also been investigated in various coronaviruses in ORF1a and ORF1b, as well as in short ORFs coding for spike, envelope glycoprotein, membrane protein, and nucleocapsid. There are no approved drugs or vaccines for human coronaviruses yet. With alarmingly increasing damage caused by COVID-19 to human lives and global economy, there is a pressing need for its prevention and treatment strategies through vaccines or potential drugs. This knowledge on direction of mutational pressure may help design more effective vaccines and nucleoside analogs. If one knows the direction of mutational pressure in regions that code for neutralizing epitopes, then fragments of coding regions with the least number of mutable nucleotides in non-synonymous sites should be chosen for vaccine development. In this case the chance of immune escape will be minimal, since antibodies will be developed against the less mutable epitopes.

## Materials and Methods

### Data/Nucleotide Sequence Retrieval

The nucleotide sequences of ORF1ab from genomes of 49 coronaviruses were retrieved from GenBank. These include only reference genomes from different species of Alphacoronaviruses, Betacoronaviruses, Gammacoronaviruses, and Deltacoronaviruses including coronaviruses that caused an epidemic in 2003 (SARS-CoV), in 2013 (MERS-CoV), and an ongoing pandemic that started in December 2019 (SARS-CoV-2), available through NCBI Taxonomy data base. The IDs of those genomic sequences in GenBank are provided in the Supplementary Material.

### Nucleotide Usage Bias Analysis

The nucleotide usage biases along the length of ORF1ab from each virus were calculated in sliding windows 150 codons in length, with a step of a single codon. The “VVTAK SW” algorithm (chemres.bsmu.by) was used for the calculations. Among other indices, the algorithm calculates nucleotide usage in fourfold degenerate sites, in which all nucleotide mutations are synonymous. ORF1b in each sequence was “opened up” by the addition of “A” nucleotide into the “AAA” motif of the ribosome slippage sequence. The location of this motif has been determined with the help of GenBank description of each sequence. So, the length of complete ORF1ab for SARS-CoV-2 is equal to 7097 codons. This includes ORF1a equal to 4406 codons (until the stop-codon), and the ORF1b of 2696 codons. Nucleotide usages in the fourfold degenerate sites of ORF1a and ORF1b (the one before the “AAA” motif of the ribosome slippage sequence, and the one after that motif) were compared for all 49 viruses. Coefficients of correlations between ΔU4f and ΔA4f, between ΔU4f and ΔC4f, between ΔU4f and ΔG4f, as well as between ΔA4f and ΔC4f, were calculated.

Average nucleotide usage levels in fourfold degenerate sites have been calculated in all short ORFs from 49 genomes of coronaviruses included in this study with a help of VVTAK Protective Buffer algorithm (chemres.bsmu.by). The values of nucleotide usage indices for four genes present in all 49 species of coronaviruses (spike, envelope protein, membrane protein, and nucleocapsid protein) have been compared with those for ORF1a and ORF1b with a help of two-tailed paired *t*-test.

There are eight amino acid residues (Ala, Gly, Val, Thr, Pro, Leu, Arg, Ser) that are encoded by codons with fourfold degenerate sites in their third positions. Interestingly, Leu, Arg, and Ser are encoded by six codons each, so just four of those codons contain fourfold degenerate sites. Taken together, there are 32 codons with fourfold degenerate sites in their third positions, that is one half of all possible codons. The percent of fourfold degenerate sites among sites from third codon positions is equal to 43.85% in ORF1a and 41.56% in ORF1b in SARS-CoV-2 reference genome.

### Mutational Pressure Analysis in SARS-CoV-2

Nucleotide sequences of the complete ORF1ab of SARS-CoV-2 were retrieved with the help of NCBI BLAST algorithm. The reference genome of SARS-CoV-2 (GenBank ID: NC_045512) was used as a target. 400 full length sequences of ORF1ab belonging to different isolates of SARS-CoV-2 from all over the globe were obtained from GenBank on the 15th of April 2020. Description of sequences (including their IDs in GenBank) can be found in the Supplementary Material, as well as a UPGMA dendrogram built for them using LogDet (Tamura-Kumar) method by the MEGA X software. Those sequences have been obtained from persons suffering from COVID-19. The majority of sequences are from United States, while numerous researchers from other countries have also contributed into this set.

As it was expected, all sequences belong to the same strain of SARS-CoV-2, and the maximal number of nucleotide mutations between the ORF1ab from the reference (parental) genome and its offspring is equal to seven. So, there were no recombination events with other species of Coronaviruses detected. Those mutations happened in different sites for different isolates. That is why the maximal number of mutations between two isolates (between the offspring) is equal to 13.

The numbers of sites with all possible types of nucleotide mutations in this alignment relative to the reference sequence were calculated. Similarly, the number of sites available for each type of nucleotide mutation was calculated in the reference sequence. The rate of nucleotide mutation of a certain type is equal to the number of sites with a given mutation divided by the total number of nucleotides available for this kind of mutation. For example, the rate of C to U mutations is equal to the number of sites with C to U mutations over the number of C residues. The rates of mutations have been compared with each other with the help of unpaired *t*-test.

The rates of synonymous and non-synonymous mutations for C to U mutations were calculated. The sites in which C to U mutation is synonymous and those in which it is non-synonymous in the reference ORF1ab sequence of SARS-CoV-2 were determined. To calculate the rate of synonymous C to U transitions, the number of sites with (non)synonymous C to U mutations were divided by the number of sites in which C to U mutations are (non)synonymous. The rates of mutations for ORF1a and ORF1b were calculated separately.

### Analysis of ORF1a MERS Fragments With Different U4f Levels

To check the origin of a fragment of ORF1a from MERS (Betacoronavirus England 1) with relatively decreased U4f we performed nucleotide BLAST analysis of that fragment (codons 1400 – 2600) and the remaining fragment of ORF1a in the 3′ direction with relatively higher U4f (codons 2601 – 4460). There were 29 sequences of complete genomes of related Betacoronaviruses found with a help of “more dissimilar sequences (discontiguous megablast)” search for both sequences. Sequences that belong to the same species (MERS related Betacoronaviruses) were excluded from the search. Two alignments have been made (for each fragment of ORF1a) using a PAM (Point Accepted Mutation) method from MEGA X program. Phylogenetic trees (UPGMA dendrograms) have been constructed based on amino acid distances calculated by the LogDet (Tamura-Kumar) method and checked by the bootstrap method. Bootstrap consensus trees are provided in the Supplementary Material, as well as names and accession numbers of sequences.

### Statistical Analysis

The Student’s *t*-test was used for calculating the statistical significance: we used paired or unpaired *t*-test, were it was appropriate. *P*-values < 0.05 were considered as statistically significant. Coefficients of correlation have been calculated using MS Excel.

## Results

### Mutational Pressure in SARS- CoV-2 ORF1ab

As on the 15th of April 2020, a total of 400 full-length sequences of ORF1ab belonging to different isolates of SARS-CoV-2 were available in GenBank. The first full-length genome of SARS-CoV-2 appeared in GenBank toward the end of December 2019, followed by the other sequences. Therefore, the first reported genome of the virus can be considered as the initial (parental) sequence, while the others can be regarded as its offspring that mutated during the ongoing pandemic. If each offspring sequence is compared with the initial one, the number of mutations may seem to be relatively small (up to 7). However, when all those 400 sequences are considered, there are already 250 sites with mutated nucleotides (ignoring ambiguous results of sequencing) in this rather long ORF that consists of 21227 nucleotides.

To find out the preferable direction of those nucleotide mutations, the numbers of sites with each type of nucleotide substitution were calculated and divided by the usage of a corresponding nucleotide in a reference sequence (Khrustalev et al., 2019). Calculated frequencies of nucleotide mutations were compared with each other. Since there is a ribosome slippery sequence in the ORF1ab, we performed calculations separately for ORF1a and ORF1b, before and after the slippery site, respectively. There is a clear and strong mutational U-pressure in both ORFs (Table 1). The most frequent type of nucleotide mutation in SARS-CoV-2 ORF1ab during 4 months of the pandemic was cytosine to uracil transition (C to U). The rate of this mutation was found to be more than 6 times higher than the rate of an opposite U to C mutation in ORF1a and more than four times higher in ORF1b. The difference between the rate of C to U transitions and the rate of U to C transitions is significant (*P* < 0.05) for both ORFs. Interestingly, the rate of C to U transitions is also significantly (1.7 times) higher in the ORF1a than in ORF1b. The most frequent cause of this kind of mutation is cytosine deamination, the product of which is uracil. This mutation may be spontaneous or enzymatic. In the latter case, RNA-editing enzymes from APOBEC family may be responsible for it (Sharma and Baysal, 2017; Smith, 2017). These enzymes bind single-stranded viral RNA and deaminate cytosine residues. Spontaneous deamination occurs via oxidation of cytosine by free radicals (Gros et al., 2002). The rate of this process is higher for single-stranded RNA than for double-stranded RNA (Hendriks et al., 2010).

**TABLE 1:** Rates of nucleotide mutations (in substitution per site with a corresponding nucleotide) in ORF1ab of SARS-CoV-2: before (ORF1a) and after (ORF1b) the ribosome slippage sequence.

Type of mutation:	C→U	U→C	A→G	G→A	C→A	A→C
ORF1a	**0.0356223**	**0.005379**	0.004306	0.007928	0.001288	0.000760
ORF1b	**0.0212164**	**0.004971**	0.003633	0.001900	0.001414	0.000404
Type of mutation:	G→U	U→G	A→U	U→A	G→C	C→G
ORF1a	**0.0049075**	**0.000000**	0.000760	0.000935	0.000378	0.000429
ORF1b	**0.0082331**	**0.000382**	0.000404	0.001147	0.000633	0.000707

*Significant changes between opposite types of mutations are shown by bold font, significant changes in rate of the same type of mutation between two ORFs are shown in underlined font.*

Since ORF1a is translated more frequently than ORF1b, the rate of C to U transitions should be higher in ORF1a because of co-translational deamination of unwound RNA. Those mutations are controlled by negative selection, and we confirm this statement in the next section. Indeed, the process of translation often ends near the ribosome slippery sequence at a stop codon if the ribosome fails to slip to the -1 reading frame (Baranov et al., 2005). Therefore, ORF1b is not unwound as frequently as ORF1a. The difference between the rates of G to A and A to G transitions was not found to be significant for both ORFs. However, the rate of G to A transitions themselves is significantly higher in ORF1a than in ORF1b (Table 1). The rate of G to U transversions is significantly higher than the rate of U to G transversions in both ORFs (Table 1). The rate of G to U transversions is, of course, significantly lower than the rate of C to U transitions. However, both of these mutations do contribute to the mutational U-pressure in this viral gene. The rates of other transversions are rather low (Table 1). Their preferable directions can be determined only after some time when the virus acquires more mutations. Even now, after the first 4 months after the breaking of the interspecies barrier between pangolin and human by this virus (if the hypothesis of its appearance is correct), it is clear that the most frequent mutations in its ORF1 are C to U transitions.

### The Rate of Synonymous C to U Mutations Is Higher Than the Rate of Non-synonymous Ones in SARS-CoV-2 ORF1ab

The number of C to U mutations observed in SARS-CoV-2 ORF1ab is enough to consider the type of natural selection. To make such conclusions, the numbers of sites with synonymous and non-synonymous C to U mutations in ORF1a and ORF1b were calculated (Chi et al., 2015). After that, we divided those numbers by the numbers of sites for synonymous and non-synonymous C to U mutations in corresponding ORFs of the reference SARS-CoV-2 genome. A comparison of those rates is given in Table 2. As evident from Table 2, the rate of synonymous mutations of C to U direction is significantly higher than the rate of non-synonymous mutations of the same direction in both ORFs. The rate of synonymous C to U mutations is similar in both ORFs. In contrast, the rate of non-synonymous C to U mutations is significantly higher in ORF1a than in ORF1b.

**TABLE 2:** Description of C to U transitions in ORF1ab of SARS-CoV-2: before (ORF1a) and after (ORF1b) the ribosome slippage sequence.

	ORF1a		ORF1b	
	Synonymous	Non-synonymous	Synonymous	Non-synonymous
Number of sites with C to U mutation	32	51	17	13
Number of available sites	630	1700	403	1011
Rate of mutations, substitution per site	**0.050794**	**0.03000**	**0.042184**	**0.012859**

*Significant changes between rates of synonymous and non-synonymous mutations are shown by bold font, significant changes in rate of the same type of mutation between ORFs are shown in underlined font.*

The number of sites for synonymous C to U mutations is 2.7 (ORF1a) and 2.5 (ORF1b) times lower than the number of sites for non-synonymous C to U mutations. So, the increase of the rate of C to U mutations in ORF1a relative to ORF1b is caused by the increase of the rate of non-synonymous mutations. The reason for this growth maybe both in the higher rate of their occurrence and in the weaker negative selection. Indeed, proteins that are cleaved from the part of pp1ab polyprotein encoded by ORF1b (RNA-dependent-RNA polymerase and helicase, 3′-to-5′ exonuclease, endoRNAse, 2′-O-ribose methyltransferase) seem to be more conservative than proteins that are cleaved from its first part. An interesting fact is that the number of sites with synonymous C to U mutations is lower than the number of sites with non-synonymous C to U mutations (32 vs 51) for ORF1a, while for ORF1b it is vice versa (17 vs 13). As usual, these numbers must be normalized by the number of sites available for those mutations before the comparison.

### Nucleotide Usage Biases in ORF1ab of SARS-CoV-2, SARS-CoV, and MERS

**FIGURE 1:**
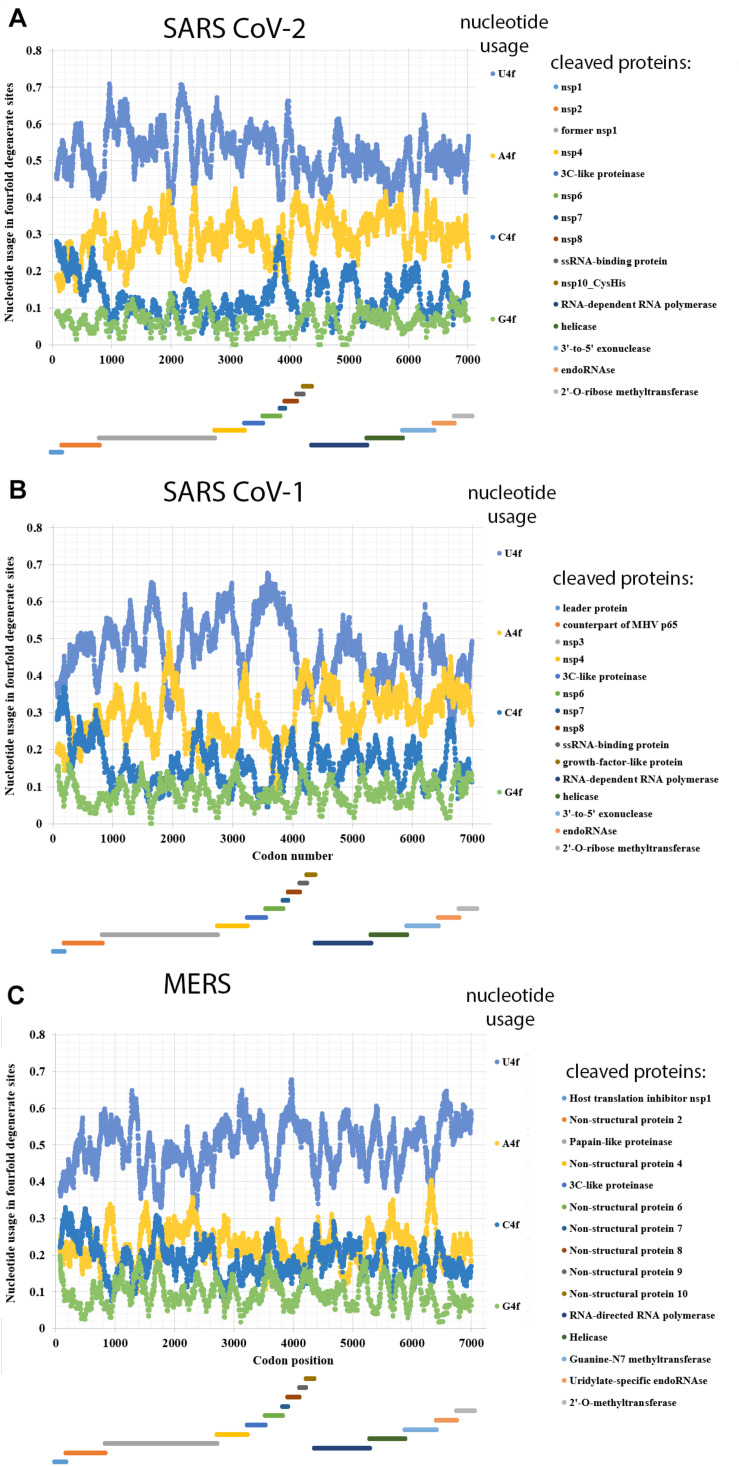
Nucleotide usage in fourfold degenerate sites along the length of ORF1ab of **(A)** SARS-CoV-2, **(B)** SARS-CoV-1, **(C)** Betacoronavirus England 1 (causative agent of MERS). The length of a sliding window is equal to 150 codons. Borders of proteins (according to the uniport annotations) that are cleaved from the long polypeptide are shown at the bottom of each graph, the border between ORF1a and ORF1b is shown as the start of a new stairway-like sequence of proteins (at the bottom). U4f is the usage of uracil in fourfold degenerate sites; A4f is the usage of adenine in fourfold degenerate sites; C4f is the usage of cytosine in fourfold degenerate sites; G4f is the usage of guanine in fourfold degenerate sites.

Nucleotide usage biases in genes are formed during long-term process of fixation of certain nucleotide mutations in numerous generations (Sueoka, 2002). As one can see in Figure 1A, the usage of uracil in fourfold degenerate sites of ORF1ab from SARS-CoV-2 is quite high: 53.6 ± 0.2% in the ORF1a and 49.0 ± 0.2% in the ORF1b implying that the rates of C to U transitions and G to U transversions have been much higher than the rates of opposite mutations for a very long time in predecessors of the current virus. Nowadays, almost one-half of nucleotides in fourfold degenerate sites of SARS-CoV-2 ORF1ab are uracil residues. Interestingly, in ORF1b, the usage of uracil is still significantly lower than in the ORF1a. It means that the tendency observed during the mutagenesis of SARS-CoV-2 in human cells is the same as during its mutagenesis in cells of its former hosts.

Adenine is the second most common/frequent nucleotide to occur in the fourfold degenerate sites (Figure 1A). This shows that C to U transitions are frequent in RNA minus strands of the virus as well. However, they are not as frequent as those in its RNA plus strands. This fact may be interpreted as the evidence that viral RNA plus strand is a target for APOBEC editing soon after the entry into the host cell. If an infection is successful, coronavirus suppresses expression of host proteins with its early protein nsp1 (Shen et al., 2019), and its RNA minus strands formed later are not edited by APOBEC. The level of A4f is significantly higher in the ORF1b than in ORF1a.

Both cytosine and guanine are extremely rare in fourfold degenerate sites of SARS-CoV-2 ORF1ab (Figure 1A). However, there are still several areas with C4f level around 20% both in ORF1a and ORF1b.

In the reference strain of SARS-CoV-1 (responsible for the 2002–2003 SARS epidemic), the difference in nucleotide usage levels between ORF1a and ORF1b is even more noticeable than in that from the reference strain of SARS-CoV-2 (Figure 1B). In ORF1a, the level of U4f is 49.3 ± 0.2%, while in ORF1b, it is 42.9 ± 0.2%. The values of U4f for SARS-CoV-1 ORF1ab are significantly lower than those for SARS-CoV-2. As to the values of A4f, they are equal to 26.7 ± 0.2 and 32.7 ± 0.2% for SARS-CoV-1 and 28.3 ± 0.2 and 31.5 ± 0.2% for SARS-CoV-2 ORF1a and ORF1b, respectively. So, the magnitude of change in A4f usage before and after the ribosome slippage site is higher for SARS-CoV-1 than for SARS-CoV-2. It means that one may expect the highest rate of successful ribosome slippage for evolutionary predecessors of SARS-CoV-2 than for those of SARS-CoV-1.

Nucleotide usage biases in fourfold degenerate sites along the ORF1ab of one of the MERS viruses (namely, in Betacoronavirus England 1 strain) are shown in Figure 1C. The usage of U4f is distributed almost identically along the length of ORF1a (49.2 ± 0.2%) and ORF1b (50.5 ± 0.2%). A slight decrease of U4f in ORF1a is because of the area between codons #1400 and #2600, where U4f is lower than in other parts of the same ORF. The difference between A4f in ORF1a and ORF1b is insignificant (22.4 ± 0.1 and 22.2 ± 0.2%). There are areas with relatively elevated C4f usage in this ORF, while G4f is always somewhere near the point of 10%. Due to the absence of changes in nucleotide usage biases before and after the ribosome slippage sequence, one may speculate that this event (ribosome slippage) was almost always successful for evolutionary predecessors of the MERS virus, while the lack of difference in U4f between ORF1a and ORF1b is more likely the consequence of a recombination event (see “Discussion” section). It is essential to check whether MERS virus is rather an exception or a rule among different coronaviruses.

### Nucleotide Usage Biases Along the Length of ORF1ab From Alpha-, Beta-, Gamma-, and Deltacoronaviruses

Nucleotide usage biases along the length of ORF1ab have been determined in 46 more completely sequenced coronaviruses. From the data given in Table 3, it can be concluded that there is a mutational U-pressure in all those known species. However, the average value of U4f in fourfold degenerate sites varies from 39 to 71%, while the average value of A4f varies from 16 to 40%. Once again, U4f is always significantly higher than A4f in all species of coronaviruses, in both ORF1a and ORF1b. If we take all 49 species of coronaviruses together, U4f is significantly higher in ORF1a than in ORF1b, while A4f and C4f are significantly lower in ORF1a than in ORF1b. The level of G4f is the same in both ORFs. A similar trend is observed when only Alphacoronaviruses or Betacoronaviruses are considered. However, in Deltacoronaviruses, the differences in U4f and C4f between two ORFs are still significant, while the difference in A4f is not significant.

**TABLE 3:** Average values of nucleotide content in fourfold degenerate sites for ORF1a and ORF1b (ORF1ab after the ribosome slippage sequence) for 49 species of coronaviruses.

	ORF1a				ORF1b			
Species of viruses	A4f	U4f	G4f	C4f	A4f	U4f	G4f	C4f
**Betacoronaviruses**								
SARS-CoV-2	0.282559	**0.536080**	0.058141	0.123220	0.314966	**0.489943**	0.065602	0.129489
Bat coronavirus RaTG13	0.279333	**0.538640**	0.057880	0.124147	0.317106	**0.492413**	0.063180	0.127301
SARS type 1	0.267316	**0.492572**	0.080709	0.159403	0.326612	**0.429282**	0.084896	0.159210
Betacoronavirus England 1	0.223760	**0.492141**	0.096139	0.187959	0.222440	**0.505384**	0.087213	0.184963
MERS	0.223758	**0.489458**	0.097238	0.189546	0.221945	**0.506858**	0.086123	0.185074
Bat coronavirus HKU4-1	0.243970	**0.549858**	0.086605	0.119567	0.247374	**0.527550**	0.098643	0.126433
Bat coronavirus HKU5-1	0.219651	**0.430946**	0.126756	0.222647	0.233393	**0.428189**	0.137513	0.200905
Bat coronavirus HKU9-1	0.205231	**0.517617**	0.143563	0.133589	0.248432	**0.471643**	0.135637	0.144288
Bat Hp-betacoronavirus Zhejang 2013	0.259312	**0.449471**	0.127087	0.164130	0.280761	**0.428425**	0.109822	0.180992
Bovine coronavirus TCG-19	0.210180	**0.594721**	0.101922	0.093176	0.229055	**0.512969**	0.110493	0.147483
China rattus coronavirus HKU24	0.220346	**0.470419**	0.147173	0.162062	0.244320	**0.445679**	0.136765	0.173236
Betacoronavirus Erinaceus	0.294150	**0.482614**	0.109619	0.113616	0.303022	**0.478903**	0.082157	0.135918
Human coronavirus HKU1	0.207154	**0.701287**	0.037482	0.054077	0.217268	**0.643761**	0.049733	0.089239
Human coronavirus OC43	0.212476	**0.590722**	0.09732	0.099481	0.237404	**0.514365**	0.101690	0.146541
Mouse hepatitis virus	0.188042	**0.448827**	0.166824	0.196306	0.216405	**0.421702**	0.166198	0.195695
Rabbit coronavirus HKU14	0.196470	**0.590026**	0.112055	0.101449	0.252936	**0.497622**	0.100746	0.148696
Rat coronavirus Parker	0.186994	**0.459502**	0.167466	0.186037	0.214727	**0.438168**	0.157116	0.189988
Rousettus bat coronavirus	0.240135	**0.400558**	0.175626	0.183681	0.262846	**0.396113**	0.177551	0.163490
**Alphacoronaviruses**								
Bat coronavirus 1A	0.197439	**0.592162**	0.064526	0.145873	0.201381	**0.610753**	0.061812	0.126054
Bat coronavirus CDPHE15USA2006	0.211126	**0.559267**	0.087088	0.142519	0.192782	**0.557644**	0.087228	0.162345
Bat coronavirus HKU2	0.166628	**0.652947**	0.062911	0.117514	0.199027	**0.584848**	0.083031	0.133093
BtMr-AlphaCoV SAX2011	0.209349	**0.556779**	0.097402	0.136470	0.238494	**0.507276**	0.081311	0.172918
BtNv-AlphaCoVCS2013	0.161644	**0.585640**	0.111608	0.141108	0.177368	**0.559410**	0.106309	0.156914
BtRf-AlphaCoV HuB2013	0.245029	**0.558607**	0.084154	0.112210	0.261599	**0.490377**	0.101753	0.146271
BtRf-AlphaCoVYN2012	0.180421	**0.662049**	0.054639	0.102891	0.205510	**0.596575**	0.06731	0.130605
Camel alphacoronavirus	0.208933	**0.593785**	0.076436	0.120846	0.235353	**0.546779**	0.088768	0.129099
Human coronavirus 229E	0.196892	**0.597574**	0.083607	0.121927	0.227798	**0.552015**	0.084688	0.135499
Human coronavirus NL63	0.190087	**0.712752**	0.034993	0.062168	0.205129	**0.677292**	0.036428	0.081151
Lucheng Rn rat coronavirus	0.184384	**0.557929**	0.083031	0.174656	0.193101	**0.527395**	0.094169	0.185335
Mink coronavirus	0.228678	**0.546826**	0.102932	0.121563	0.249230	**0.535591**	0.099763	0.115417
NL63-related Bat coronavirus	0.189823	**0.577504**	0.079057	0.153616	0.210809	**0.551366**	0.075071	0.162754
Porcine epidemic diarrhea virus	0.199831	**0.522930**	0.096686	0.180553	0.191929	**0.514827**	0.105637	0.187607
Rousettus bat coronavirus HKU10	0.207802	**0.592856**	0.091049	0.108293	0.209272	**0.557211**	0.083908	0.149609
Scotophilus bat coronavirus 512	0.201188	**0.544340**	0.098665	0.155807	0.203487	**0.569018**	0.082646	0.144849
Swine enteric coronavirus	0.240513	**0.573303**	0.070222	0.115962	0.257887	**0.516850**	0.086973	0.138291
Transmissible gastroenteritis virus	0.246219	**0.576135**	0.057783	0.119863	0.270000	**0.524111**	0.082884	0.123004
Wencheng Sm shrew coronavirus	0.264157	**0.548777**	0.091945	0.095121	0.250293	**0.609173**	0.086062	0.054471
**Gammacoronaviruses**								
Avian infectious bronchitis virus	0.280215	**0.514909**	0.105775	0.099101	0.272677	**0.507519**	0.096646	0.123157
Beluga whale coronavirus	0.251623	**0.483031**	0.137046	0.128301	0.258106	**0.472517**	0.137313	0.132064
**Deltacoronaviruses**								
Bulbul coronavirus HKU11	0.271396	**0.538218**	0.074722	0.115664	0.273612	**0.512611**	0.079387	0.134389
Common-moorhen coronavirus	0.280305	**0.604411**	0.059284	0.056000	0.245981	**0.611189**	0.064450	0.078381
Magpie-robin coronavirus HKU18	0.160454	**0.413506**	0.141714	0.284326	0.199450	**0.391917**	0.128339	0.280294
Munia coronavirus HKU13	0.271725	**0.430193**	0.116227	0.181855	0.281493	**0.422466**	0.100486	0.195556
Night-heron coronavirus HKU19	0.355703	**0.449160**	0.068657	0.126479	0.362609	**0.398874**	0.078126	0.160391
Porcine coronavirus HKU15	0.241219	**0.471506**	0.105017	0.182258	0.267771	**0.423215**	0.101061	0.207953
Sparrow coronavirus HKU17	0.227610	**0.427294**	0.111387	0.233708	0.251201	**0.419277**	0.109639	0.219882
Thrush coronavirus HKU12	0.266167	**0.564516**	0.071252	0.098065	0.276805	**0.503051**	0.098791	0.121352
White-eye coronavirus HKU16	0.270696	**0.499818**	0.072899	0.156586	0.255200	**0.502311**	0.08055	0.161940
Wigeon coronavirus HKU20	0.270351	**0.483598**	0.111698	0.134354	0.259563	**0.486631**	0.106387	0.147419

*The value of U4f is written in bold font in case if it is significantly higher than A4f, G4f, and C4f from the same ORF.*

There are several exceptions from the rule among studied viruses, in which the value of U4f is significantly lower in ORF1a than in ORF1b. They are 3 out of 19 Alphacoronaviruses; 2 out of 18 Betacoronaviruses; 3 out of 10 Deltacoronaviruses (Table 3). There are at least two causes of the existence of those exceptions. At first, coronaviruses are prone to recombination with each other and with other viruses (Su et al., 2016). That is how some fragments of ORF1ab may be exchanged during recombination, and a newly acquired fragment of ORF1ab will possess an outstanding bias in fourfold degenerate sites. After a certain number of generations, nucleotide usage levels in fourfold degenerate sites will become almost identical through the whole length of ORF1a and ORF1b again. From this point of view, there may be such half-homogenized fragment in the ORF1a of MERS (Figure 1C). The “homogenization” of biases in twofold degenerate sites from third codon positions takes longer time than their “homogenization” in fourfold degenerate sites (Khrustalev et al., 2020). Biases in first and second codon positions will be “improved” after an even more extended period of time (Khrustalev et al., 2012).

**FIGURE 2:**
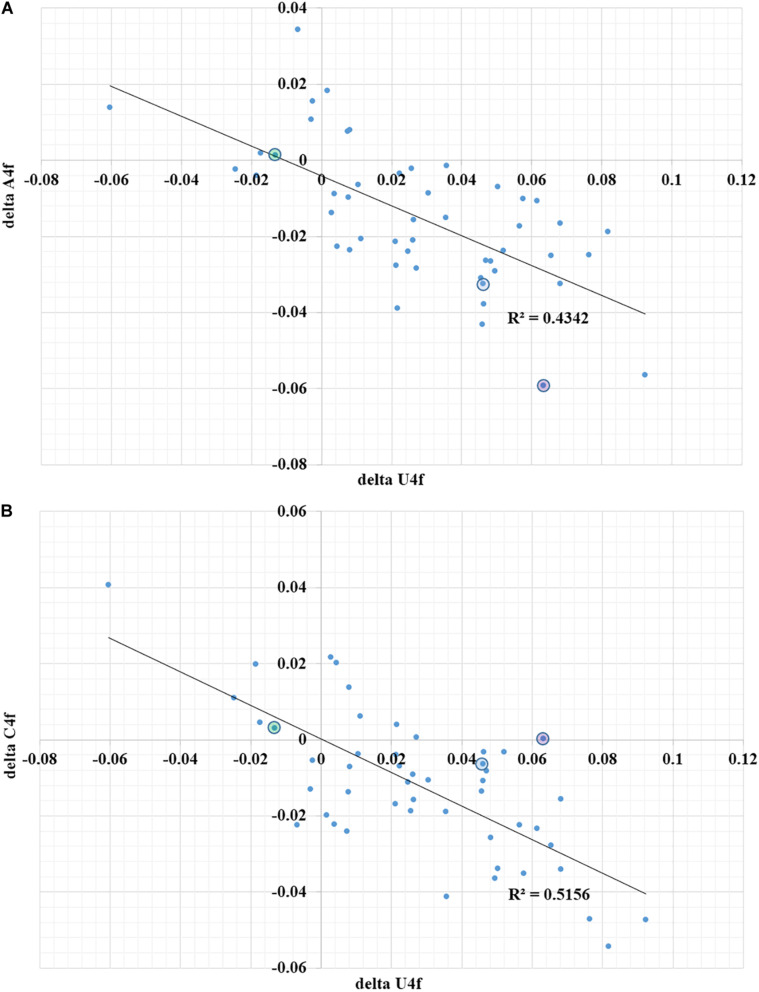
The dependence of the difference in A4f **(A)** and C4f **(B)** on the difference in U4f between ORF1a and ORF1b for 49 species of coronaviruses. Dots corresponding to SARS-CoV-2 are in blue circles, dots corresponding to SARS-CoV-1 are in violet circles, dots corresponding to Betacoronavirus England 1 (MERS) are in green circles. U4f is the usage of uracil in fourfold degenerate sites; A4f is the usage of adenine in fourfold degenerate sites; C4f is the usage of cytosine in fourfold degenerate sites.

Second, the quality of a regulatory element responsible for ribosome slippage should be different in different coronaviruses (Baranov et al., 2005). As one can see in Figure 2A, the difference in average values of U4f in two parts of ORF1ab may reach more than 8%, or it may be equal to just 1%. Interestingly, there is a correlation between the difference in U4f and the difference in A4f between two parts of ORF1ab (*R* = −0.66). Indeed, the higher the difference in U4f, the higher (by module) the difference in A4f. The same relationships are there between the difference in U4f and the difference in C4f between two parts of ORF1ab (*R* = −0.72). The difference in A4f, however, shows no correlation on the difference in C4f (*R* = 0.11). It means that in some viruses, U4f in the ORF1a is increased mostly because of the decrease of A4f, while in others, it is increased mostly because of the decrease of C4f. Both SARS-CoV-2 and SARS-CoV-1 belong to the group in which U4f is growing mostly because of the decrease of A4f. The chance of success for ribosome slippage is one of the key factors of viral pathogenesis (Plant et al., 2013). According to Figure 2A, evolutionary predecessors of SARS-CoV-2 were likely to be able to synthesize more RNA-dependent-RNA-polymerase at the onset of infection than those of SARS-CoV-1.

### Nucleotide Usage Biases in Short ORFs of Alpha-, Beta-, Gamma-, and Deltacoronaviruses

Short ORFs situated downstream of the long ORF1ab of coronaviruses are quite variable. There are just four out of thirty-eight described ORFs that are mapped and named in all species of coronaviruses. The purpose of those small ORFs is always under question, since in most of the cases they are predicted by a gene finding software, and the presence of corresponding proteins is not confirmed experimentally. One of the criterions for their description in GenBank reports is the presence of specific transcription start sites upstream (Zúñiga et al., 2004). Those sites are quite sensitive to mutations in regions of RNA situated nearby (Sola et al., 2005), and they can easily mutate themselves, since the core of transcription regulatory sequence contains two residues of Cytosine (ACGAAC). For example, there is ORF10 described for SARS-CoV-2 in the 3′ end of the genome. The same ORF exists in the closest relative of SARS-CoV-2 that has been found earlier in bats (Bat coronavirus RaTG13), while it is not described in the GenBank report as protein coding sequence. Homologous sequence is there in the genome of SARS-CoV-1 as well, but the reading frame contains a single stop codon in the middle. So, we analyzed just permanent ORFs encoding spike (surface) glycoprotein (S), envelope protein (E), membrane glycoprotein (M), and nucleocapsid phosphoprotein (N).

For all the 49 species of coronaviruses taken together, the level of U4f in the spike ORF is significantly higher than the level of A4f (51.78 ± 2.33% vs 22.93 ± 1.37%, respectively, *P* < 0.001). So, there is mutational U-pressure in the spike gene as well. The level of U4f in spike ORF is significantly lower than the one in ORF1a (53.53 ± 2.01%), but the difference is not significant if we compare it with ORF1b (50.75 ± 1.86%). The level of C4f is significantly higher in spike ORF, than in both ORF1a and ORF1b (16.46 ± 1.44; 13.96 ± 1.26; 15.16 ± 1.08%, respectively), if we compare all the viral species together. The values of A4f, U4f, G4f, and C4f for spike ORF of each studied species are provided in the Supplementary Material.

Gene coding for envelope protein is also enriched by uracil residues in its fourfold degenerate sites. However, the level of U4f in that short ORF (41.59 ± 3.22%) is significantly lower than in both ORF1a and ORF1b. At the same time, levels of A4f, G4f, and C4f are significantly higher inside envelope protein ORF, than in both ORF1a and ORF1b.

The same tendency is there in membrane protein and nucleocapsid phosphoprotein ORFs. The level of U4f is still the highest one (40.08 ± 2.41 and 46.02 ± 2.45%, respectively), but the levels of C4f (19.98 ± 1.57 and 20.71 ± 1.45%, respectively), are getting close to the values of A4f (25.87 ± 1.88 and 24.23 ± 1.55%, respectively).

Mutational U-pressure is seen in all the four short permanent ORFs. Since corresponding mRNAs are transcribed from small subgenomic RNA minus strands, one cannot judge the influence of their expression levels on nucleotide usage biases. All mutations that happen in both minus and plus strands of subgenomic RNAs are not directly inherited by viral offspring. Since small ORFs are not translated from genomic RNA plus strands, and there is still mutational U-pressure in them, one can conclude that the most of C to U mutations are not happening during translation, but during replication. On the other hand, the values of U4f in 3 out of 4 small ORFs are significantly lower than in both ORF1a and ORF1b, while levels of C4f are significantly higher in all of them. It means that mutational U-pressure existing in the whole genome of each coronavirus is enhanced in those regions that serve not just as genomic RNA, but also as mRNA at the early steps of infection.

## Discussion

Determination of the mutational pressure direction should be a starting point in any vaccine design study (Khrustalev et al., 2020). If the most frequent type of nucleotide mutation is known, one may try to choose “cold” spots for this mutation as targets for future vaccine development instead of “hot” spots. Based on our study, we suggest that fragments of RNA of SARS-CoV-2 that have a higher level of U in first and second codon positions and a higher level of C in synonymous sites for C to U mutations should be chosen for vaccine designing.

It has been shown that the genome of SARS-CoV-2 is subject to nucleotide usage bias toward A + U in its ORFs, including ORF1 (Kandeel et al., 2020; Sheikh et al., 2020). In this study, we examined the usage of U and A separately, since A = U and G = C parity rules do not work in these viruses. Nucleoside analogs have been suggested as anti-COVID-19 drugs (Agostini et al., 2019). Based on the results of this study, uracil analogs would be more effective in treating COVID-19 than analogs of cytosine and guanine nucleosides. Adenine analogs should be capable of effectively inhibiting the synthesis of RNA-minus strands. Therefore, they may also prove to be good therapeutic strategy but mostly during the early stages of infection on a cellular level, when A-rich RNA minus strands are synthesized. Though care should be taken that any nucleoside analog used to treat COVID-19 wouldn’t be recognized by the proof-reading machinery of the virus (Agostini et al., 2019) or the drug will be rendered ineffective.

Sequences obtained from all over the world belong to active viruses that are under the control of negative selection, which can be confirmed by the fact that the rate of non-synonymous mutations is less than the rate of synonymous ones. Indeed, the virus is not under strong immune pressure or the pressure of antiviral drugs at the moment. These stresses are known to cause diversifying (positive) selection of non-synonymous mutations (Nijmeijer and Geijtenbeek, 2019). On one hand, mutagenesis of C to U causes amino acid substitutions that can lead to quite drastic consequences for the fitness of a virus. Therefore, most of them are eliminated. On the other hand, mutations of C to U direction may help in immune evasion, since amino acid replacements caused by those nucleotide substitutions are capable of destroying linear B-cell epitopes more than other types of single nucleotide mutations (Khrustalev, 2010). The reason of this phenomenon is in the properties of genetic code. Namely, C to U transitions cause a lot of substitutions of residues usually situated on a surface of a protein to residues usually buried in its core, such as: Pro to Leu; Ser to Phe and Leu; Thr to Ile and Met; His to Tyr; Arg to Cys and Trp. Because of such replacements linear B-cellular epitopes are becoming shorter since their surface accessibility decreases (Zúñiga et al., 2004).

Nsp1 is a non-structural protein present in Alpha- and Betacoronaviruses, but not in Gamma- and Deltacoronaviruses (Shen et al., 2019). Nsp1 can inhibit the expression of host proteins (Huang et al., 2011; Tanaka et al., 2012). Likely, the expression of non-specific antiviral enzymes belonging to APOBEC and ADAR families are also inhibited by nsp1. If most of the C to U mutations in ORF1a are caused by APOBEC editing of viral RNA during initial steps of infection, then it may be expected that there will be no difference in nucleotide usage levels in fourfold degenerate sites between ORF1a and ORF1b in Gamma- and Deltacoronaviruses. But, as evident from Table 3, the above-mentioned differences still exist in most of the Gamma and Deltacoronaviruses, implying that a significant fraction of C to U mutations is not caused by APOBEC editing, but by oxidative deamination of cytosine residues.

The existence of RNA exonuclease (nsp14) in genomes of coronaviruses may be the cause of the observed mutational U-pressure. If during the post-replicational proof-reading most of the deaminated and oxidized nucleotides are removed, the only product of deamination remaining is a canonical amine base, uracil. As inosine (product of adenine deamination) is a non-canonical amine base, it is removed during proof-reading, whereas, uracil (product of cytosine deamination) being a canonical amine base, is not removed, as proof-reading activity effectively removes mismatched pairs of canonical nucleotides, pairs of canonical nucleotides with non-canonical ones, but it cannot recognize U that has already appeared in place of C on a matrix strand before the replication as a mutated nucleotide. Interestingly, some non-canonical amine bases, like 8-oxo-G (the product of guanine oxidation), can somehow still pass through the proofreading machinery, as several G to U transversions have been detected in this study.

It was shown that coronaviruses that lack their proof-reading machinery accumulate non-canonical nucleotides much better than when functional proof-reading machinery is present (Smith et al., 2013). Indeed, 5-formyl-uracil is paired with adenine in the absence of nsp14 in SARS-CoV-1 and MERS viruses, and it leads to the increase of U to C and A to G transitions since this non-canonical nucleotide can pair with G even better than with A (Smith et al., 2013). So, in the absence of proof-reading, the overall rate of mutations becomes higher, while the bias in their rates becomes weaker.

Taken together, coronaviruses are well known for their low mutation rates achieved due to proof-reading during RNA replication (Bouvet et al., 2012). However, they still cannot repair C to U transitions with this mechanism, as U is a canonical amine base. Moreover, C to U transitions occur before the replication, and the resulting U makes a correct pair with A during the complementary RNA strand synthesis. Therefore, mutational U-pressure is seen in all coronaviruses throughout the whole length of their genomic RNA.

The effectiveness of frameshifting for ORF1ab for different coronaviruses has been reported to be in the range of 20 – 45% (Baranov et al., 2005). For the Mouse hepatitis virus A59, the rate of effective frameshifting is higher (from 48 to 70%) (Irigoyen et al., 2016). For SARS-CoV-1, this rate has been reported as 17.5%, (Baranov et al., 2005). In infectious bronchitis virus, the rate of successful frameshifting is 30 – 40% (Brierley et al., 1989; Dinan et al., 2019). For human coronavirus 229E the rate of successful frameshifting is about 20 – 30% (Herald and Siddell, 1993). Hence, it may be speculated that highly efficient frameshifting leads to the decrease of ΔU4f, and low rate of effective frameshifting causes the increase of ΔU4f, while there is a lack of data on frameshifting effectiveness obtained in the same laboratory by the same method to check this hypothesis.

Here we should state that for all coronaviruses just short sequences of RNA containing ribosome slippery motif and some cis-acting elements as well were studied being expressed in a vector in a certain cell line. Conditions in which the effectiveness of frameshifting has been determined were quite different in different laboratories. That is why one cannot be sure that those determined values are comparable with each other. Also, some trans-acting elements from the rest of those genomes may be responsible of the increase or the decrease of frameshifting success rate. Recently, sequences of frameshift elements have been compared for SARS CoV-1 and SARS CoV-2 (Kelly et al., 2020). Since those sequences showed the same rate of successful frameshifting in the same conditions, it is likely that trans-acting motifs and other factors are responsible of the difference in frameshifting success rates between evolutionary predecessors of those viruses. Another factor that can influence the rate of frameshifting success is the cell type: in HEK293T cells it was around 20% for both SARS CoV-1 and SARS CoV-2 sequences, but in HeLa cells it was around 30% (Kelly et al., 2020). So, the host may be the key factor that determines the effectiveness of frameshifting and so the magnitude of the difference in U4f levels between ORF1a and ORF1b for different species of coronaviruses.

The effectiveness of frameshifting for MERS virus is equal to 14% (Hu et al., 2016), while the level of U4f inside its ORFa is lower than the one in its ORFb. This fact does not support the abovementioned hypothesis. To check another hypothesis that recombination events may disturb the distribution of U4f between ORF1a and ORf1b, we performed BLAST search using the nucleotide sequence coding for the fragment between codons 1400–2600, and the nucleotide sequence coding for the remaining part of the ORF1a of Betacoronavirus England 1 (MERS) starting from the codon 2601. There are 29 genomes of Betacoronaviruses that demonstrate similarity with these two sequences revealed by “discontiguous megablast.” For both amino acid sequences the closest relatives are the same: 4 samples of betacoronaviruses from bats (the closest one is Bat coronavirus isolate NC_034440.1 from Uganda). Differences appear at the longer evolutionary distance. Amino acid sequence of MERS encoded by a fragment with lower U4f (codons 1400 – 2600) demonstrates high similarity with five sequences that belong to Hedgehog coronavirus 1 and its relatives, while other 20 sequences are grouped together on the separate branch of a dendrogram (see Supplementary Material). In contrast, sequences of Hedgehog coronavirus 1 and its relatives are outgroups in a dendrogram built for the amino acid sequence encoded by the fragment with a higher U4f (codons 2601 – 4460), while 20 other sequences (including Bat coronavirus HKU4-1) are grouped together with MERS sequence. These data show that recombination event with one of the Hedgehog betacoronaviruses happened not recently, but before the divergence of MERS with its four closest relatives.

Amine (nitrogenous) bases of both DNA and RNA are prone to oxidative damage (Gros et al., 2002). That damage results in the appearance of purine and pyrimidine derivatives that are not included in the set of four major amine bases, except the situation with cytosine deamination in RNA leading to the appearance of uracil. It has been proven that the rate of guanine oxidation is approximately 2 times higher if guanine is situated in the single stranded fragment of DNA, than in the double stranded one (Joffe et al., 2003). For cytosine deamination in DNA it has been experimentally proven that cytosine is deaminated approximately 140-fold more slowly when present in the double helix than in the single stranded DNA (Frederico et al., 1990). DNA turns to the unwound single stranded state during replication and transcription, while RNA turns to that state during replication and transcription (in viruses with RNA genomes), and also during translation. During replication leading and lagging strands are replicated in different way, and so they accumulate mutations differently, forming characteristic replication-associated nucleotide usage bias (Lobry and Sueoka, 2002; Sueoka, 2002). During transcription transcribed and non-transcribed strands also exist in different conditions, and so accumulate mutations in different way, producing well known transcription-associated nucleotide usage bias (Polak et al., 2010). The same situation is expected in RNA that has regions that are translated at a different frequency and so being unwound (Qu et al., 2012) during shorter or longer periods of time.

Exactly in genomes of Coronaviruses we found out that their overall U4f bias is stronger in highly translated ORF1a than in less frequently translated ORF1b, and in short ORFs that are not translated from the genomic RNA plus strand at all. So, it is likely that “additional” C to U transitions are happening during translation of genomic RNA plus strands.

One of the multiple consequences of mutational U-pressure is the decrease of secondary structure amount in RNAs. However, this decrease is not so strong, as in case of A-pressure, since uracil residues frequently make non-canonical base pairs with G residues (Sato et al., 2009). It was shown that RNA with greater secondary structure is expressed more efficiently because structure increases mRNA half-life while having no effect on translation efficiency (Mauger et al., 2019). From this point of view, relative increase in U4f in ORF1a should not affect the speed of its translation, while it should result in the increased chance of its oxidative damage when it is not translated, compared to ORF1b and small ORFs. In other words, the increase in U4f may in its turn lead to the higher chance of remaining cytosine residues deamination. However, according to our hypothesis, the initial factor that causes increased rate of C to U transitions in ORF1a relative to ORF1b and small ORFs should be the increased rate of translation, while non-translated parts of genomic RNA plus strand are protected from cytosine deamination better, since they are not translated at all.

## Conclusion

In this study we proved that the rate of C to U transitions and the intensity of U-bias in fourfold degenerate sites are higher in ORF1a of coronaviruses, situated before the ribosome slippery sequence, than in the less frequently translated ORF1b which is situated after the slippery sequence. Moreover, U-bias is weaker in small ORFs that are not translated from genomic RNA plus strands. So, the overall U-pressure observed in all the examined ORFs from different species of coronaviruses is a consequence of proof-reading allowing C to U transitions to happen during replication, while in ORF1a efficiently translated from genomic RNA plus strands U-pressure is stronger.

## Data Availability Statement

All datasets presented in this study are included in the article/Supplementary Material.

## Author Contributions

VK and RG: conception and design. VK and TK: data acquisition, analysis, interpretation of the data, and writing of the manuscript. VK, RG, TK, AS, VP, and SK: reviewing and writing of the manuscript. All authors contributed to the article and approved the submitted version.

## Conflict of Interest

The authors declare that the research was conducted in the absence of any commercial or financial relationships that could be construed as a potential conflict of interest.

## Supplementary Material

TABLE S1 Average values of nucleotide usage in genes coding for spike, envelop, membrane, and nucleocapsid proteins from 49 species of coronaviruses; the dendrogram representing ORF1ab sequences from 400 isolates of SARS CoV-2; dendrograms confirming recombination event in MERS predecessor.
